# Laparoscopic Management of Primary Omental Torsion

**DOI:** 10.1155/2021/5536178

**Published:** 2021-02-24

**Authors:** Alin Mihețiu, Alexandra Sandu, Dan Bratu, Cristina Mihețiu

**Affiliations:** ^1^“Lucian Blaga” University of Sibiu, County Clinical Emergency Hospital, Sibiu, Romania; ^2^“Lucian Blaga” University of Sibiu, Romania; ^3^Centre Hospitalier Sud Francilien, Corbeil-Essonnes, France

## Abstract

Primary omental torsion is an unusual condition, known for its rarity and for the particularity of being intraoperatively diagnosed, in nearly all cases. At the clinical evaluation, this pathology commonly mimics other etiologies of acute abdomen. Hemoperitoneum and necrosis of the omentum are rarely associated with the omental torsion, but when the association is found, then it means that the vascular injuries are irreversible and the required surgical procedure may be far more complex than simple devolvulus. In search of the treatment of choice, laparoscopy proved its effectiveness as a diagnostic and therapeutic tool, while the open surgery approach can be described in many cases as being too invasive. A 37-year-old female patient presented with the generic symptoms of acute appendicitis. Surgical treatment was initiated. During laparoscopy, the abdomen was attentively explored, highlighting the presence of a twisted omentum with hemoperitoneum and necrosis. Omental excision and peritoneal drainage were performed. The evolution was favorable. Another check-up was done at 6 months postoperatively, displaying no signs or symptoms of relapse.

## 1. Introduction

Eitel made the first description of this disease, in 1899. He stated that the omentum, also known as epiploon, twists along its own axis. This mechanism can be the main cause of other physiopathological changes which can range from simple edema to ischemia or necrosis. Nearly 300 cases of greater omentum torsion have been described in the literature [1–3].

Omental torsion is classified as primary or secondary. The primary torsion of the omentum is due to a mobile, thickened segment which revolves around a fixed, proximal point and appears in the absence of any inflammatory or secondary intraabdominal pathology. Secondary torsion of the omentum implies the existence of underlying pathology and most commonly occurs around two fixed points (bipolar fixation) [1, 4].

It is notable that symptoms fail to prove any specificity. Torsion of the omentum is a rare entity, which often presents itself as an acute abdomen, with clinical findings that resemble symptoms associated with appendicitis.

Preoperative diagnosis depends on and is warranted by the characteristic appearance of omental torsion on ultrasound and CT. In most cases, epiploon torsion is an intraoperative surprise. In the absence of a primary laparoscopic approach, laparotomy is needed as a method of surgical treatment.

## 2. Case Report

We present the case of a 37-year-old overweight female patient, with no previous records of any health concerns or surgical interventions. The patient presented with abdominal pain of 36-hour duration. Initially, the pain was diffused and mainly located in the mesogastrium but then progressively moved to the right iliac fossa. The pain was accompanied by nausea and two episodes of vomiting. Physical examination of the abdomen revealed tenderness with signs of peritoneal irritation in the right iliac fossa.

At the admittance, blood investigations showed leukocytosis with a total leukocyte count of 14 820/mmc, 10,91 neutrophils/mmc, and mild anemia that was indicated by the slightly low values of hemoglobin, 11,8 g/dl, and hematocrit, 36,1% (normal values range between 37% and 47%).

Clinical and paraclinical investigations indicated the preoperative diagnosis of acute abdomen, raising the suspicion of acute appendicitis. Abdominal ultrasound revealed free fluid filling the pouch of Douglas, without changes that could suggest any other condition than the one initially suspected. Therefore, a CT scan was not performed, as it was not considered a routine test in presumed cases of acute appendicitis. After establishing the diagnosis of acute surgical abdomen, surgery was performed by laparoscopic approach detecting hemoperitoneum (approximately 500 ml hemorrhagic liquid) located in the lesser pelvis, interhepatophrenic, and in the right paracolic gutter.

**Figure 1:**
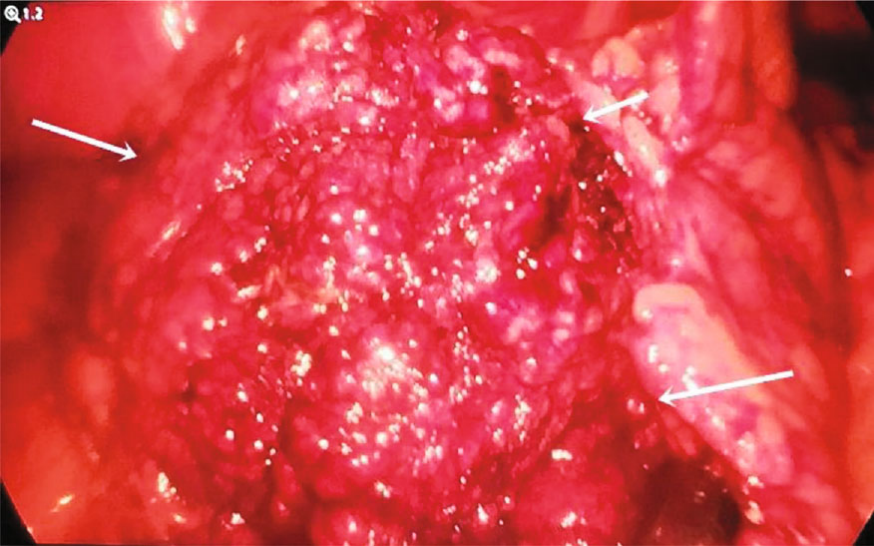
Omental mass - laparoscopic view.

**Figure 2:**
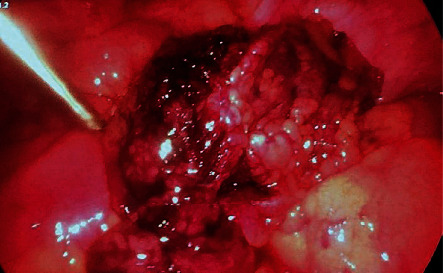
Omental mass - laparoscopic view.

At greater omentum level, a tumor mass with inflammatory changes and a contorted base was found (Figures 1 and Figures 2).

**Figure 3:**
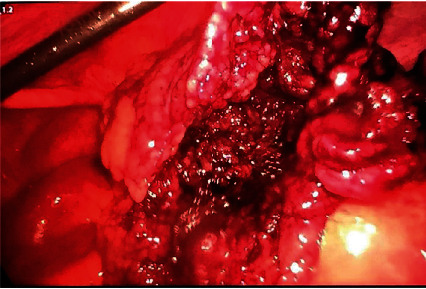
Omental mass dissection.

**Figure 4:**
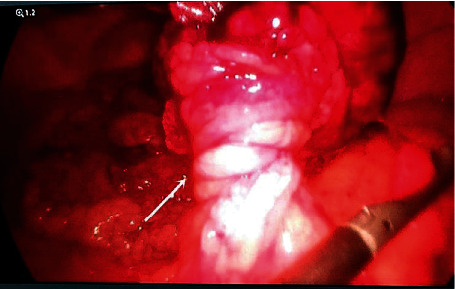
Omental torsion.

During the dissection process, extensive hemorrhagic and focal necrosis areas were noticed inside the formation (Figure 3). The patient underwent laparoscopic omentectomy, starting from the contorted area (Figure 4).

The resected specimen was removed using an endobag, through the trocar insertion site (10 mm) from the left iliac fossa. In order to remove the omental segment, the incision is required to be enlarged by 2,5 more centimeters. Subsequently, peritoneal lavage and drainage of the Douglas pouch were performed.

Drain tubes were suppressed on the 2^nd^ day postoperatively, and after 3 days of hospital stay, the patient was discharged.

Histopathology report suggested twisted omental segment measuring 21 × 10 × 4 cm with pieces of hemorrhagic and necrotic tissue. Microscopic examination highlighted adipose tissue consisting of adipocytes with marked infiltrate, hematous in appearance, necrosis, and minimal chronic inflammatory infiltrate. The examined material lacked atypical findings.

The follow-up was scheduled with a reassessment at 1 month and 6 months postoperatively that showed a favorable evolution with no further changes or complications.

## 3. Discussion

Torsion of the epiploon is a rare clinical event, difficult to diagnose preoperatively because it mimics other causes of acute surgical abdomen. It is frequently confused with acute appendicitis due to clinical resemblance, being remarked in less than 4 per 1,000 cases of presumed appendicitis (incidence 0,0016–0,37%) [5–7].

Males are more frequently affected (sex ratio 2 : 1). It usually manifests in patients between 20 and 50 years old [1].

The greater omentum develops embryologically from the dorsal mesoderm, and it is made up of four layers that are anchored by the great gastric curvature, transverse colon, and neighboring organs, also passing in front of the small intestines. Its left border continues with a distinct structure, gastrolienal ligament and descends inferiorly with the splenocolic ligament. The blood supply of the greater omentum is provided by the right and left gastroepiploic arteries which traverse its layers.

The most commonly reported torsional mechanism is a rotation around the vascular axis represented by the right gastroepiploic artery. The explanation is owed to the greater mobility of the right omental segment compared to the left one, which is well-fixed because of gastrosplenic and splenocolic ligaments [7–9].

The classification criteria divide the epiploon torsion in primary and secondary, respectively, in unipolar and bipolar. In the unipolar torsion, the proximal part is fixed; the rotation occurring around it and the other parts are free and known as “tongues.” The primary torsion is always unipolar. Bipolar torsion involves the existence of two fixed points of the omentum: distal and proximal. The secondary torsion can be both unipolar and bipolar [10, 11].

Having distinguished primary from secondary omental torsion, it remains to distinguish their etiopathogenetic theories. The agents considered as causing secondary omental torsion are represented by preexisting abdominal pathologies such as abdominal tumors, cysts, inflammation, adhesion syndromes, or hernias. The etiopathogenesis of primary torsion is still debatable [1, 12].

A classification into predisposing factors and precipitating factors has been proposed [13].

Obesity, irregular omental pad, abnormal vascular source, bifid or accessory omentum, tongue-like projections, or other anatomical variations are recognised as predisposing factors [1, 14].

Precipitating factors are abdominal trauma, sudden changes in body positions, hypoperistalsis, or binge eating [5].

In a physiopathological manner, the torsion of the proximal omental part initially compromises the venous return causing congestion, inflammation, edema, and hemorrhagic extravasations (hemoperitoneum), leading to arterial ischemia and resulting in tissue necrosis.

The symptoms are inconclusive. The most usual symptom is pain in the right iliac fossa or right hypochondrium. Nausea, vomiting, loss of appetite, and fever may appear inconsistently. The occurrence of leukocytosis, a high level of C-reactive protein, and even hyperbilirubinemia, probably through hemolysis mechanisms, are regularly met [15].

Differential diagnoses are acute appendicitis, acute cholecystitis, mesenteric lymphadenitis in children, Meckel's diverticulum, gynecological disorders, perforated ulcer, or acute pancreatitis.

Ultrasonography is proved to be useful in guiding the preoperative diagnosis, as it can evidentiate fluid in the peritoneal cavity and less often a hyperechoic solid mass with irregular hypoechoic edges, adhering to the abdominal wall [9, 16].

A CT scan can be more accurate, showing an ovoid mass in the umbilical region or in the right abdomen. The tomographic feature for epiploic torsion is the evidence of a conglomerate consisting of adipose tissue together with a fibrous whirl. The coexistence of the vascular pedicle and the “whirlpool” signs can guide the radiologist toward the diagnosis of epiploon torsion. Nonetheless, omental torsion is not infrequently mistaken for lipoma, liposarcoma, appendicitis, intestinal volvulus, and panniculitis [17, 18].

The potential effectiveness of conservative management with antibiotics and anti-inflammatory medication is controversial, as shown in the surgical literature. Conservative treatment predisposes to local complications, abscesses, adhesion syndromes, or even to the omission of an underlying condition that can be masked by the symptoms and imaging data of an epiploon torsion. It is possible, but unlikely, for omental torsion to resolve spontaneously [4, 19].

Surgical treatment consisting of segmental omentectomy can be performed by the laparotomy or laparoscopic approach. Owing to the rarity of this condition and the lack of specific symptoms that can lead to a mistaken diagnosis, omental torsion can be confused with acute appendicitis. The perfect resemblance between these two entities may be the reason why surgical teams cannot resist the impulse to place the first incision in the right iliac fossa, subsequently performing midline laparotomy.

By 2015, less than 300 cases of epiploon torsion were described in the literature, of which 26 were approached laparoscopically [4, 8, 9].

In our attempts to find out the most preferred and rational approach for patients who suffer from omental torsion, we conducted a research from 2015 to 2020, using PubMed and Scholar databases.

At the time of this investigation, querying the terms “primary omental torsion,” “omental torsion,” “laparoscopy,” and “laparotomy” returned 23 cases of primary omental torsion between 2015 and 2020. Pediatric cases and those with no relevance to the subject were excluded. No language exclusion criteria were used. The average age of patients with this pathology was 41,5 years with a male predominance revealed by a 3 : 1 sex ratio (M : F = 3 : 1). Right lower quadrant abdominal pain appeared as the main complaint, found in 69,5% of cases.

In 17 cases, laparoscopy was the selected surgical procedure and patients underwent laparoscopic omentectomy associated, in 3 of the cases, with laparoscopic appendectomy.

In 3 other cases, McBurney's incision was used to proceed with the surgery, which at that time was thought to be an appendectomy. Later on, a midline incision was used for a better exposure of the abdominal cavity. Another 3 cases were primarily approached by open surgery (midline laparotomy). During intraoperative examination, epiploon necrosis was found in 91,3% of cases, accompanied by hemoperitoneum or serohemorrhagic liquid in 4 of these cases. Except for one particular case, which presented fever and postoperative intra-abdominal fluid collection that was conservatively resolved, no further complications were noticed (Table 1).

Unfortunately, it was not possible to find out exactly the duration of hospital stay for each patient. However, considering the average length of hospitalization for open approach in surgery, it is generally believed that those who were subjected to laparoscopy tend to be discharged sooner than those who underwent laparotomy.

In addition to the 26 laparoscopically treated cases mentioned in the literature up to 2015, our research identified 17 more cases until the present moment. Without adding the case presented above to the previous ones, we point out that 43 cases of laparoscopically treated primary omental torsion have been described.

Laparoscopy is currently the procedure of choice for epiploon torsion. In the vast majority of cases, there is no need for intraoperative conversion to laparotomy because the removal of the resected specimen can be done with a minimum widening of the trocar sites.

The attitude towards the appendix is debatable. The only reasonable consideration for proceeding with appendicectomy may be the possibility to exclude appendicitis from the differential diagnosis of an upcoming abdominal condition. However, being given the current situation with no appendicular inflammatory signs, we considered that the correct surgical option should not include appendicectomy.

Its role in immune regulation of bacterial flora, as well as its absence correlated with inflammatory bowel disease, affections of the heart, or Parkinson's disease, demonstrates that the vermiform appendix is far from being a purposeless organ [41–43].

Laparoscopy, as an ever-expanding approach, allows diagnostic accuracy, good view of the affected omentum, thus avoiding unnecessary operations, immediate and distant complications of open surgery, and prolonged hospitalization. Also, a better social and professional reintegration might be achieved.

## 4. Conclusion

Primary torsion of the omentum is a rarely met condition, its symptoms mimicking an acute surgical abdomen. Extensive imaging studies can aid in diagnosis and management and can help with deciding the most appropriate treatment solution. Laparoscopic surgery offers multiple advantages including its minimal invasivity and exhibits higher diagnostic accuracy of the rare forms of acute abdomen. Due to laparoscopy, patients are exempt from the consequences and complications of open surgery.

## Figures and Tables

**Table 1:** Primary omental surgical approach between 2015 and 2020—literature review.

	Year	Authors	Patient age	Gender	Main symptoms		Surgical approach	Intraoperative findings	Postoperative evolution
1	2015	Zaleta-Cruz et al. [8]	24	M	Right quadrant	Lower pain	Laparoscopic omentectomy, appendectomy	Omental necrosis	Fever. Intraperitoneal collection treated conservatively
2		Hussain et al. [20]	22	M	Right quadrant	Lower pain	Laparoscopic omentectomy	Omental necrosis	Uneventful
3		Alexiou et al. [21]	52	W	Right quadrant	Lower pain	McBurney incision, midline laparotomy, omental resection	Omental congestion	Uneventful
4			68	M	Right quadrant	Lower pain	Laparotomy, omental resection	Omental congestion	Uneventful
5		Shinde et al. [22]	45	M	Abdominal	Right side pain	Laparoscopic omentectomy	Omental necrosis	Uneventful
6		Konkena et al. [23]	24	M	Right abdominal	Upper pain	Laparoscopic omentectomy, appendectomy	Omental necrosis	Uneventful
7		Umale et al. [24]	39	M	Right quadrant	Lower pain	Laparotomy, omentectomy	Omental necrosis	Uneventful
8		Ahmed et al. [25]	41	W	Right quadrant	Lower pain, anemia	Laparotomy, omentectomy	Omental necrosis	Uneventful
9		Agarwal et al. [26]	40	M	Right quadrant	Lower pain	Laparotomy, omentectomy	Omental necrosis	Uneventful
10	2016	Kumar et al. [27]	47	M	Lower abdominal pain		Laparotomy, omental resection	Omental necrosis	Uneventful, discharge the third postoperative day
11		Raza et al. [28]	26	W	Pelvic pain		Laparoscopic omentectomy	Omental necrosis	Uneventful, discharge the third
12		Kolandaivelu et al. [29]	55	W	Abdominal	Right side pain	Laparotomy, omental resection	Omental necrosis	Postoperative day
13		Yu et al. [30]	43	W	Right quadrant	Lower pain	Laparoscopic omentectomy	Omental necrosis	Uneventful
14		Dhooghe et al. [31]	67	W	Right quadrant	Upper pain	Laparoscopic omentectomy	Omental necrosis	Uneventful
15		Cremonini et al. [32]	28	M	Right quadrant	Lower pain	Laparoscopic omentectomy	Omental necrosis	Uneventful
16	2018	Karanikas et al. [33]	31	M	Right quadrant	Lower pain	McBurney incision, laparotomy, omentectomy, appendectomy	Omental necrosis, hemoperitoneum	Uneventful, discharge the fifth postoperative day
17		Johri et al. [34]	38	M	Right quadrant	Lower pain	Laparoscopic omentectomy, appendectomy	Omental necrosis, hemoperitoneum	Uneventful
18		Kafadar et al. [35]	45	M	Right quadrant	Lower pain	Laparoscopic omentectomy	Omental necrosis	Uneventful
19			27	M	Right quadrant	Lower pain	McBurney incision, midline laparotomy, omentectomy, appendectomy	Omental necrosis hemoperitoneum	Uneventful
20		Khalid et al. [36]	19	M	Epigastric and right quadrant	Lower pain	Laparoscopic omentectomy	Omental necrosis	Uneventful
21	2019	Kataoka et al. [37]	50	M	Right quadrant	Upper pain	Laparoscopic omentectomy	Omental necrosis	Uneventful
21		Gupta et al. [38]	26	M	Right quadrant	Lower pain	Laparoscopic omentectomy, appendectomy	Omental necrosis, hemoperitoneum	Uneventful
22	2020	Pawar and Kishore [39]	38	M	Right abdominal	Side pain	Laparoscopic omentectomy	Omental necrosis	Uneventful
23		Bolat and Teke [40]	61	M	Right quadrant	Lower pain	Laparotomy, omentectomy	Omental necrosis	Uneventful

## References

[1] Andreuccetti J., Ceribelli C., Manto O., Chiaretti M., Negro P., Tuscano D. (2011). Primary omental torsion (POT): a review of literature and case report. *World Journal of Emergency Surgery*.

[2] Eitel G. G. (1899). Rare omental torsion. *New York Medical Record*.

[3] Morris J. H. (1932). Torsion of the omentum. *Archives of Surgery*.

[4] Occhionorelli S., Zese M., Cappellari L., Stano R., Vasquez G. (2014). Acute abdomen due to primary omental torsion and infarction. *Case Reports in Surgery*.

[5] Scabini S., Rimini E., Massobrio A. (2011). Primary omental torsion: a case report. *World Journal of Gastrointestinal Surgery*.

[6] Itenberg E., Mariadason J., Khersonsky J., Wallack M. (2010). Modern management of omental torsion and omental infarction: a surgeon’s perspective. *Journal of Surgical Education*.

[7] Pinedo-Onofre J. A., Guevara-Torres L. (2007). Omental torsion. An acute abdomen etiology. *Gaceta Médica de México*.

[8] Zaleta-Cruz J. L., Rojas-Méndez J., Garza-Serna U., González-Ruvalcaba R., de Elguea-Lizarraga J. O., Flores-Villalba E. (2017). Omental torsion. Case report. *Cirugía y Cirujanos*.

[9] Silva E., Carvalho A. F., Rocha D. (2014). Omental whirl associated with bilateral inguinal hernia: a case report. *Journal of Medical Case Reports*.

[10] Joshi S., Cuthbert G. A., Kerwat R. (2016). Omental torsion, a rare cause of acute abdomen. *British Medical Journal Case Reports*.

[11] Tsironis A., Zikos N., Bali C., Pappas-Gogos G., Koulas S., Katsamakis N. (2013). Acute abdomen due to primary omental torsion: case report. *The Journal of Emergency Medicine*.

[12] Katagiri H., Honjo K., Nasu M., Fujisawa M., Kojima K. (2013). Omental infarction due to omental torsion. *Case Reports in Surgery*.

[13] Adams J. T. (1973). Primary torsion of the omentum. *The American Journal of Surgery*.

[14] Leitner M. J., Jordan C. G., Spinner M. H., Reese E. C. (1952). Torsion, infarction and hemorrhage of the omentum as a cause of acute abdominal distress. *Annals of Surgery*.

[15] Nishiwaki S., Yomoda D., Iizuka K., Naitou K., Yoshida M., Ida T. (2007). A case of torsion of the omentum secondary to a recurrent inguinal hernia. *Journal of Japan Surgical Society*.

[16] Oğuzkurt P., Kotiloğlu E., Tanyel F. C., Hiçsönmez A. (1995). Primary omental torsion in a 6-year-old girl. *Journal of Pediatric Surgery*.

[17] Naffaa L. N., Shabb N. S., Haddad M. C. (2003). CT findings of omental torsion and infarction: case report and review of the literature. *Clinical Imaging*.

[18] Coppin T., Lipsky D. (2016). Twisting and infarction of the entire greater omentum managed by laparoscopy: a report of two cases. *Acta Chirurgica Belgica*.

[19] Coulier B. (2006). Segmental omental infarction in childhood: a typical case diagnosed by CT allowing successful conservative treatment. *Pediatric Radiology*.

[20] Hussain K., Munir A., Wahla M. S., Mian M. A., Masood J. (2015). Laparoscopic management of primary segmental omental infarction mimicking acute appendicitis. *Journal of the College of Physicians and Surgeons–Pakistan*.

[21] Alexiou K., Ioannidis A., Drikos I., Sikalias N., Economou N. (2015). Torsion of the greater omentum: two case reports. *Journal of Medical Case Reports*.

[22] Shinde J., Pandit S., Fernandes A., Joshi V., Naik R. (2015). Case report of primary omental torsion. *Medical Journal of Dr. D.Y. Patil University*.

[23] Kollu S., Neelam P., Konkena J. R., Gunta S. R. (2015). Torsion omentum mimicking appendicitis: a rare cause of pain abdomen. *Journal of Dr. NTR University of Health Sciences*.

[24] Umale N., Dagwar A., Tiwari S. (2015). Omental torsion: a diagnostic challenge. *International Surgery Journal*.

[25] Ahmed A., Jabbour G., Zitoun A. (2015). Anemia as one of presenting symptoms in an adult with cyst and torsion of the omentum - a case report. *Chirurgia*.

[26] Agarwal S., College G. G. M., Sir J J Group of Hospitals, Mumbai A. S., Tayade M. B. (2015). Primary omental infarction presenting as a parietal wall swelling: a rare case report. *Journal of Medical Science and Clinical Research*.

[27] Kumar A., Shah J., Vaidya P. (2016). Primary omental gangrene mimicking appendicular perforation peritonitis--a case report. *International Journal of Surgery Case Reports*.

[28] Raza N., Kania P., Bhamare P. (2016). A rare case of omental torsion - a surprise diagnosis of acute pelvic pain. *International Journal of Reproduction, Contraception, Obstetrics and Gynecology*.

[29] Kolandaivelu P. G., Lakshmana R., Balamurugan R., Arun P. S. (2016). Primary omental torsion - a rare case report. *IAIM*.

[30] Yu J. S., Lee W. S., Kim Y. H. (2016). Primary torsion of lesser omentum presented with acute abdomen and successfully managed with laparoscopic surgery. *Chinese Medical Journal*.

[31] Dhooghe V., Reynders D., Cools P. (2016). Torsion of a bifid omentum as a rare cause of acute abdomen: a case report. *Journal of Medical Case Reports*.

[32] Cremonini C., Bertolucci A., Tartaglia D., Menonna F., Galatioto C., Chiarugi M. (2015). Acute abdomen caused by greater omentum torsion: a case report and review of the literature. *Ulusal Travma ve Acil Cerrahi Dergisi*.

[33] Karanikas M., Kofina K., Ali F. B. (2018). Primary greater omental torsion as a cause of acute abdomen-a rare case report. *Journal of Surgical Case Reports*.

[34] Johri V., Dhaduk V., Mushtaque N., Jain N., Reddy P. K. (2018). Role of laparoscopy as diagnostic and therapeutic tool in management of omental torsion. *Sri Lanka Journal of Surgery*.

[35] Kafadar M. T., Cetinkaya I., Bardakci O. (2018). Primary omental torsion mimicking acute appendicitis: an unusual cause of acute abdominal pain in a young male. *Eurasian Journal of Emergency Medicine*.

[36] Khalid M. A., Amin A., Amir M. (2018). Torsion of lesser omentum: a rare presentation of acute abdomen. *Journal of Shifa Tameer-e-Millat University*.

[37] Kataoka J., Nitta T., Ota M. (2019). Laparoscopic omentectomy in primary torsion of the greater omentum: report of a case. *Surgical Case Reports*.

[38] Gupta R., Farhat W., Ammar H. (2019). Idiopathic segmental infarction of the omentum mimicking acute appendicitis: a case report. *International Journal of Surgery Case Reports*.

[39] Pawar N., Kishore A. (2020). Primary omental infarction: a rare cause of acute abdomen. *International Surgery Journal*.

[40] Bolat H., Teke Z. (2020). Primary omental torsion with massive necrosis A case of uncommon surgical emergency. *Annali italiani di chirurgia*.

[41] Girard-Madoux M. J. H., de Agüero M. G., Ganal-Vonarburg S. C. (2018). The immunological functions of the appendix: an example of redundancy?. *Seminars in Immunology*.

[42] Vitetta L., Chen J., Clarke S. (2019). The vermiform appendix: an immunological organ sustaining a microbiome inoculum. *Clinical Science*.

[43] Cosentino M., Comi C., Marino F. (2019). The vermiform appendix in Parkinson’s disease: at the crossroad of peripheral immunity, the nervous system and the intestinal microbiome. *Autoimmunity Reviews*.

